# Editorial: Involvement of Blood Brain Barrier Efficacy, Neurovascular Coupling and Angiogenesis in the Healthy and Diseased Brain

**DOI:** 10.3389/fphys.2021.771069

**Published:** 2021-10-26

**Authors:** Daniela Carnevale, Fabrice Dabertrand, Clotilde Lecrux, Jean-Luc Morel

**Affiliations:** ^1^Department of Molecular Medicine, Sapienza University of Rome, Rome, Italy; ^2^Research Unit of Neuro and Cardiovascular Pathophysiology, IRCCS Neuromed, Pozzilli, Italy; ^3^Department of Anesthesiology, University of Colorado Anschutz Medical Campus, Aurora, CO, United States; ^4^Department of Pharmacology, University of Colorado Anschutz Medical Campus, Aurora, CO, United States; ^5^Montreal Neurological Institute, McGill University, Montreal, QC, Canada; ^6^Univ. Bordeaux, CNRS, IMN, UMR 5293, Bordeaux, France

**Keywords:** blood brain barrier, neurovascular coupling, neurodegenerative disorders, stroke, traumatic brain injury, pericyte

As we wrote to introduce this topic, the complexity of the neurovascular unit (NVU) ensure two crucial function's in brain: the blood brain barrier (BBB) and neurovascular coupling (NVC). In this first volume, reviews and articles, reporting original research and methods, address the theme of this Research Topic through pathologies such as stroke or traumatic brain injury (TBI) by *in vivo* or post-mortem techniques both in animal models and in humans. Original points of view are presented on the functions of pericytes, glycocalyx, and Wnt pathways, as well as on vascular alterations and their link with the loss of nervous system functions in preeclampsia, amyotrophic lateral sclerosis, and Parkinson's disease.

Single-cell omic analyses have shown a radically increased vascular diversity along the arterial-venous axis compared to our previous understanding. This diversity should be considered in studies of neurovascular functions. The diverse functions of the endothelial cells (ECs), pericytes, and vascular smooth muscle cells (VSMC) should be considered alongside the perivascular macrophages that reside in the Virchow-Robin space. Moreover, the perivascular fibroblast-like cells, identified by RNAseq on arteries and veins (but not capillaries), are also described and proposed to be the pericyte precursors. Finally, astrocytes are included in the list because of the direct interaction between their endfoot processes and the ECs. The review of all cells participating in the neurovascular unit (NVU) is a good entry into the field of NVC and to understand the complexity of the links between cerebrovascular disorders and neurodegenerative diseases (Ross et al.). The functional importance of heterogeneity of cell subtypes reported by transcriptomic analysis is illustrated here with heterogeneity of calcium responses of astrocytes induced by cortical hyperemia due to whisker stimulation (Sharma et al.). This work suggests that the heterogeneity of calcium response is able to transduce multiple specific signals to fine-tune the NVC process.

The role of pericytes in NVC is under development not only because of its function in the regulation of brain flow, but also because of other currently unknown functions involved in pathologies. In fact, pericytes cover 90% of the length of capillary beds. The recent research using RNAseq approaches has proposed that several pericyte lines present in the brain that regulate cerebral blood flow also have potential other functions. The comparison of available RNAseq to characterize pericyte subtypes is exhaustively presented (Hariharan et al.). The diversity of potassium channels expressed in pericytes around brain capillaries suggest their important role in metabolism-electrical coupling required for cerebral blood flow (CBF) regulation. Moreover, the putative roles of TRP-, voltage-gated calcium- and chloride channels, as well as the reticulum calcium channels inositol trisphosphate receptors (InsP3R) are also discussed. The regulation of these channels is assumed by the variety of G protein-coupled receptors (GPCRs) expressed in pericytes in response to external stimuli (Hariharan et al.). As presented in other articles (see below), disorders affecting brain function could be linked to alteration of the NVU and especially pericytes. As mentioned in the perspectives in Hariharan et al., “pericytes appear exceptionally sensitive to pathological perturbations,” but the specific pharmacological targeting of ion channels and GPCRs expressed in pericytes could represent new therapeutic approaches. One research article illustrates this by the description of the role of NLRP3 on pericytes. In fact, its deficiency in transgenic mice induced a decrease in the number of pericytes as well as reduced collagen-IV platelet-derived growth factor receptor β (PDGFRβ), and CD13 expression. Moreover, in cultured pericytes, the process is inhibited by MCC950, the antagonist of NLRP3 and Il-1β treatment (Quan et al.). Research on pericytes and other cells that compose the NVU has benefited greatly from the development of technologies that allow *in vivo* monitoring of cellular activity in animal models using various techniques, including two-photon microscopy. These techniques require the installation of a cranial window according to standardized procedures (Kiliç et al.). The time-controlled focal photothrombosis has classically been used to described the acute effect of ischemia, but it can also be used to follow the time course of the BBB alterations in the penumbra and to measure the distance of effect, including the contralateral hemisphere, in order to describe the possible spatiotemporal progression of the damages correlated to the intensity of the ischemia (Weber et al.). By coupling two-photon microscopy with the use of fluorescein isothiocyanate (FITC)-gelatin (as a MMP9-activity probe) and photothrombosis (using Rose Bengal) in a mouse model expressing tdTomato in pericytes, it has been possible to follow the time course of capillary occlusion and its physiological consequences (Underly and Shih). During ischemia, the pericytes are immediately affected and participate in the protein degradation via MMP9-dependent mechanism. L-NIL (inhibitor of nitric oxide (NO) synthases) attenuated capillary leakage and the addition of anisomycin (inhibitor of protein synthesis), led to near complete elimination of FITC-gelatin cleavage and vascular leakage. These results indicate that both NO synthase and new protein synthesis are involved in the rapid activation of MMP-9 during ischemia (Underly and Shih).

Stroke is the most important acute event that induces NVU remodeling. Freitas-Andrade et al. present a comprehensive update on this remodeling. General concepts about stroke and therapeutic approaches are linked to the endocrine function of ECs to induce neuronal development. However, how neuronal activity is responsible for plasticity and remodeling of cerebrovascular network (angiogenesis and barriergenesis) has also been studied. The time course of the ischemia-reperfusion effects is important to understand the alterations of EC structure and functions; the roles of pericytes, astrocytes, microglial cells, and the perivascular macrophages' roles on the BBB and NVC efficacies. Where and when these processes are engaged is crucial to understand the importance of biological processes such as degradation of the basement membrane or angiogenesis following ischemic and hemorrhagic strokes. The authors also addressed the issue of sexual differences in cerebrovascular outcomes of stroke through the prism of the effects of hormones on CBF and risk of ischemic stroke. Finally, limitations of preclinical stroke research concerning models of stroke are discussed. This review is well-completed by another article focused on glycocalyx, which is proposed to be responsible for endothelial mechanobiology in collaboration with channels specialized in mechanosensitivity. Variations of pressure on the BBB (or cells of the NVU) is one of the pathways that signals pathological changes (Nian et al.). Collagen-IV and fibronectin could represent markers of extracellular matrix reorganization due to ischemia as shown by immunohistochemistry in preclinical animal models and in the human brain (Michalski et al.). To test a new therapeutic pathway, more than one parameter should be followed. After ischemia, DL-3n-butylphthalide promotes angiogenesis in the perifocal region to restore the vasculature, but it can also acutely protect the BBB integrity as suggested in rat models by oral administration 1 h after the beginning of reperfusion process starting 2 h from a transient middle cerebral artery occlusion (Mamtilahun et al.).

The second severe alteration to the NVU is due to TBI. The review of preclinical experiments performed on mice have shown that alterations of the BBB can trigger or enhance the neurodegenerative disorders following mild TBI (Wu et al.). Because TBI and ischemic and hemorrhagic strokes have some similarities, Menet et al. recapitulated the works on genetic variants of Wnt partners representing risk factors of stroke as well as preclinical studies on animal models reporting Wnt partners as biomarkers or targets to reduce the deleterious effects of ischemic and hemorrhagic stroke and their implications in TBI. Because Wnt pathways are involved in the regulation of neurogenesis, synaptogenesis, angiogenesis, and BBB formation and stabilization as well as in dendritic cells implicated in immunity and inflammation, they are potentially “druggable targets” (Menet et al.).

Alterations of the NVU and NVC are also observed in neurodegenerative disorders. The accumulation of amyloid peptides is considered to drive the pathogenesis of Alzheimer's disease and cerebral amyloid angiopathy (CAA). Interestingly, the amyloid precursor protein (APP) could have a role in BBB maintenance (Ristori et al.). The links between nitric oxide and amyloid pathways in ECs, vascular disorders observed in transgenic mice with APP alterations (KO, expression of mutations-induced neurodegeneration, etc.), the APP physiological role in ECs (particularly in angiogenesis even if the functionality of the new vessels is always moot), and cell adhesion and transcription factors (AICD provided from the cleavage of APP is a regulator of transcription and calcium signaling) are reviewed. CAA could also be induced by the impairment of the transport of amyloid peptides through the basement membrane, and the molecular interactions between amyloid peptides and basement membrane proteins could be responsible for their aggregation and pathogenicity (Howe et al.).

The alteration of the BBB is now well-documented in AD patients and animal models, but it is also observed in Parkinson's disease (PD) animal models. Alterations of blood pressure, angiogenesis and vascular remodeling are reported in PD patients, and now BBB disruption is reported in the white matter of PD patients by MRI study (Al-Bachari et al.).

Since the nervous and vascular systems are developed together and because of the crucial role of NVC to sense the neuronal function, it is essential to know the development of the vascular network of the spinal cord to better consider the post-traumatic repair of this nervous tissue and also to understand pathologies such as amyotrophic lateral sclerosis or arteriovenous malformations (Vieira et al.).

Finally, vascular pathologies can induce or exacerbate cognitive problems as shown with hypertension. For example, préclampsia is a common hypertensive disorder in pregnant women potentially linked with cognitive disorders. Mice, chronically overexpressing human angiotensinogen and renin (R^+^A^+^ mice), display characteristics of preeclampsia and have now been validated to investigate cognitive alterations in post-partum females and their pups (Trigiani et al.).

In conclusion, as we can read in this first volume of “Involvement of Blood Brain Barrier Efficacy, Neurovascular Coupling and Angiogenesis in the Healthy and Diseased Brain,” many questions are still open to better understanding the interactions between cells of the NVU to study the efficacy of brain functions, the activated molecular pathways, and the time course of their alterations after vascular trauma or in neurodegenerative disorders.

## Author Contributions

All authors listed have made a substantial, direct, and intellectual contribution to the work, and approved it for publication.

## Funding

FD received awards from the CADASIL Together We Have Hope non-profit organization; a research grant from the Center for Women's Health Research located at the University of Colorado Anschutz Medical Campus; a research grant from the University of Pennsylvania Orphan Disease Center in partnership with the cureCADASIL, and the National Heart, Lung, and Blood Institute R01HL136636. J-LM received grants from Center National de la Recherche Scientifique (CNRS) and Centre National des études spatiales (CNES). DC received grants from the Italian Ministry of Health (MOH) for the ricerca corrente program.

## Conflict of Interest

The authors declare that the research was conducted in the absence of any commercial or financial relationships that could be construed as a potential conflict of interest.

## Publisher's Note

All claims expressed in this article are solely those of the authors and do not necessarily represent those of their affiliated organizations, or those of the publisher, the editors and the reviewers. Any product that may be evaluated in this article, or claim that may be made by its manufacturer, is not guaranteed or endorsed by the publisher.

